# A human IgE bispecific antibody shows potent cytotoxic capacity mediated by monocytes

**DOI:** 10.1016/j.jbc.2022.102153

**Published:** 2022-06-16

**Authors:** Natasa Vukovic, Samer Halabi, Joan Salvador Russo-Cabrera, Bart Blokhuis, Pedro Berraondo, Frank A.M. Redegeld, Dietmar M.W. Zaiss

**Affiliations:** 1 Institute of Immunology and Infection Research, University of Edinburgh, Edinburgh, UK; 2 Program of Immunology and Immunotherapy, CIMA, Universidad de Navarra, Pamplona, Spain; 3 Navarra Institute for Health Research (IDISNA), Pamplona, Spain; 4 Division of Pharmacology, Faculty of Science, Utrecht Institute for Pharmaceutical Sciences, Utrecht University, Utrecht, The Netherlands; 5 Spanish Center for Biomedical Research Network in Oncology (CIBERONC), Madrid, Spain; 6 Department of Immune Medicine, University Regensburg, Regensburg, Germany; 7 Institute of Clinical Chemistry and Laboratory Medicine, University Hospital Regensburg, Regensburg, Germany; 8 Institute of Pathology, University Regensburg, Regensburg, Germany

**Keywords:** bispecific, IgE, KiH, ADCC, monocyte, immunotherapy, cancer therapy, antibody engineering, β-hex, β-hexosaminidase, ADCC, antibody-dependent cell cytotoxicity, bsAb, bispecific antibody, CHO, Chinese hamster ovary, CMFDA, 5-chloromethylfluorescein diacetate, EGFR, epidermal growth factor receptor, FACS, flow cytometry staining, FBS, fetal bovine serum, KiH, knobs-into-holes, LZ, leucine zipper, MC, mast cell, PBMC, peripheral blood mononuclear cell, PSMA, prostate-specific membrane antigen, sdAb, single domain antibody, TAA, tumor-associated antigen

## Abstract

The generation of bispecific antibodies (bsAbs) targeting two different antigens opens a new level of specificity and, compared to mAbs, improved clinical efficacy in cancer therapy. Currently, the different strategies for development of bsAbs primarily focus on IgG isotypes. Nevertheless, in comparison to IgG isotypes, IgE has been shown to offer superior tumor control in preclinical models. Therefore, in order to combine the promising potential of IgE molecules with increased target selectivity of bsAbs, we developed dual tumor-associated antigen-targeting bispecific human IgE antibodies. As proof of principle, we used two different pairing approaches - knobs-into-holes and leucine zipper–mediated pairing. Our data show that both strategies were highly efficient in driving bispecific IgE formation, with no undesired pairings observed. Bispecific IgE antibodies also showed a dose-dependent binding to their target antigens, and cell bridging experiments demonstrated simultaneous binding of two different antigens. As antibodies mediate a major part of their effector functions through interaction with Fc receptors (FcRs) expressed on immune cells, we confirmed FcεR binding by inducing *in vitro* mast cell degranulation and demonstrating *in vitro* and *in vivo* monocyte-mediated cytotoxicity against target antigen-expressing Chinese hamster ovary cells. Moreover, we demonstrated that the IgE bsAb construct was significantly more efficient in mediating antibody-dependent cell toxicity than its IgG1 counterpart. In conclusion, we describe the successful development of first bispecific IgE antibodies with superior antibody-dependent cell toxicity–mediated cell killing in comparison to IgG bispecific antibodies. These findings highlight the relevance of IgE-based bispecific antibodies for clinical application.

Antibody-based treatment in autoimmunity and cancer therapy has shown tremendous efficacy in clinical settings. Consequently, biologicals such as mAb-based molecules represent one of the fastest growing classes of drugs in recent years (1). Among them, bispecific antibodies (bsAbs) have gained significant attention. The ability of one molecule to simultaneously target two different antigens opens a wide area of therapeutic applications, with cancer currently being the most targeted disease. Most of the bsAbs being developed as anticancer drugs can be classified into two groups: (1) immune cell engagers and (2) dual antigen targeting bsAbs (2). Immune cell engagers redirect the immune response toward the target cell by forming a bridge between an immune cell and a target cell. In addition, immune cell engagers induce the crosslinking of receptors on the immune cells. This crosslinking activates cytotoxic immune pathways, leading to target cell killing. In contrast, dual antigen targeting bsAbs target two different antigens on the target cell. Thus, the selectivity of the molecules for the target cell is increased and the risk of off-target side-effects decreased (3). In addition, if designed to interfere with two different signaling pathways in the tumor cell, dual tumor-associated antigens (TAAs) targeting bsAbs can bypass the development of treatment resistance (4). One prominent example of such a synergetic effect of bispecific antibodies is the recent clinical approval of amivantamab. This bsAb targeting epidermal growth factor receptor (EGFR) and cMet showed superior clinical efficacy to EGFR blockade monotreatment (5, 6).

Dual antigen targeting bsAbs can mediate their effector function *via* several pathways, such as complement-mediated cell lysis, antibody-dependent cell cytotoxicity (ADCC), or antibody-dependent cell phagocytosis. The isotype of the antibody can skew the immune reaction toward different effector functions, and hence, the selection of the isotype can be of critical importance for the efficacy of the treatment (7). Currently, all the mAbs with marketing approval are of the immunoglobulin (Ig) G class. However, other Ig isotypes, such as IgE and IgA, are also being explored, mainly as potential anticancer drugs. For instance, in addition to their well-known role in allergies mediated *via* mast cells (MCs), IgE antibodies can also bind to and, consequently, activate tumor-associated macrophages *via* FcεRI (8). Importantly, IgE mAbs have shown superior tumor control when directly compared to IgG in preclinical models (9, 10, 11). Furthermore, a phase I clinical trial evaluating an IgE-based molecule is currently showing promising results (12).

IgE isotype offers significant therapeutic advantages over IgG. For instance, IgE binds to its FcεRI with two orders of magnitude higher affinity than the IgG to its equivalent receptor (13). Therefore, IgE can stay bound to the immune cells expressing FcεRI, such as macrophages, monocytes, and basophils, even in the absence of antigen. Consequently, IgE shows extended tissue half-life (14). Moreover, IgE has no known inhibitory receptors (13), and contrary to IgG, which binds to the suppressive FcγRIIb, IgE only binds to activating FcRs. In preclinical models, mainly myeloid cells such as macrophages were identified as IgE effector cells (11, 15, 16), and IgE has been shown to mediate both ADCC *via* FcεRI and antibody-dependent cell phagocytosis *via* FcεRII (16). In addition to this direct effector function of IgE-based tumor treatment, tumors from rats treated with IgE tumor-targeting antibodies showed increased inflammation and macrophage skewing toward an M1-like phenotype (11). These findings suggest that, in addition to mediating superior direct cytotoxicity, IgE-based treatments can furthermore modify the tumor microenvironment in a favorable way (8).

In order to combine this rather promising potential of IgE molecules in cancer treatment with increased target selectivity, we developed dual TAAs targeting bispecific IgE antibodies. As proof of principle, we used two different approaches, one based on knobs-into-holes (KiH) and the other based on leucine zipper (LZ)–mediated molecule pairing. Here, we report the successful formation of bispecific IgE molecules with preserved antigen-binding properties and a fully functional Fc tail that mediates antigen-specific MC degranulation and *in vitro* and *in vivo* cytotoxicity.

## Results

### Design, expression, and purification of IgE bsAbs

**Figure 1:**
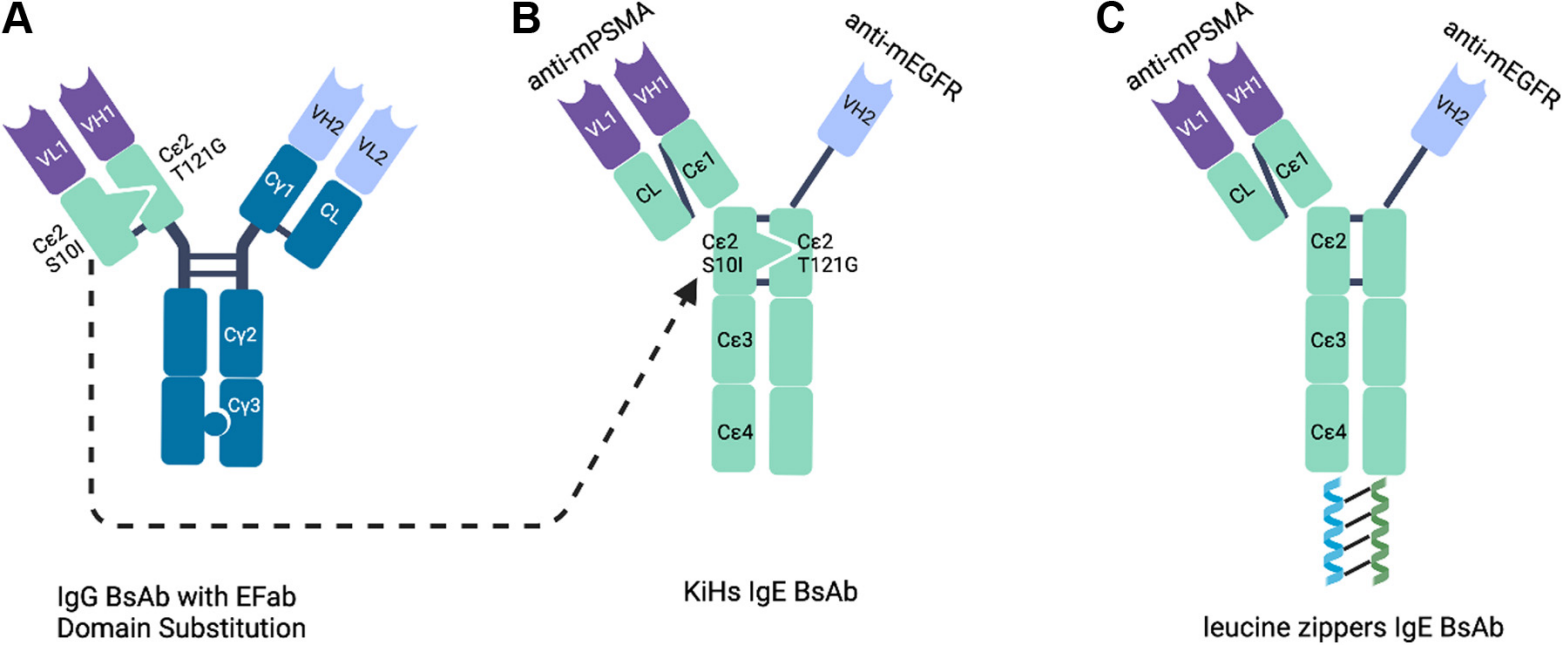
**Design of bispecific IgE molecules.***A*, IgG bispecific antibody with Efab domain substitution in which CL and Cγ1 domains in one Fab were replaced with Cε2 domains featuring KiH mutations. *B*, IgE bispecific with KiH mutations in Cε2 domains. *C*, IgE bispecific with leucine zippers. KiH, knobs-into-holes.

Interspecies differences in FcεR expression between mice and human (17, 18) exclude the use of mice and murine IgE as an adequate model for the study of IgE biology. Thus, we decided to design human bsAbs. For this purpose, two different approaches were used: (1) KiH and (2) LZs. As a proof of concept, we used in both approaches one conventional antigen-binding fragment (Fab) and a single domain antibody (sdAb) (19, 20) (Fig. 1, *B* and *C*). Because the sdAb arm does not contain a light chain, it is smaller than a conventional heavy chain. Consequently, the Fab×sdAb-Fc format offers important advantages. Firstly, due to size differences between Fab and sdAb arm, evaluation of bsAb formation can be easily done by SDS-PAGE. Secondly, any accidental light chain–heavy chain mispairing is prevented (19). As a choice of model antigens, we relied on a well-established system in our research group comprised of a conventional antibody targeting mouse prostate-specific membrane antigen (PSMA), combined with a sdAb targeting the EGFR (19, 20).

**Figure 2:**
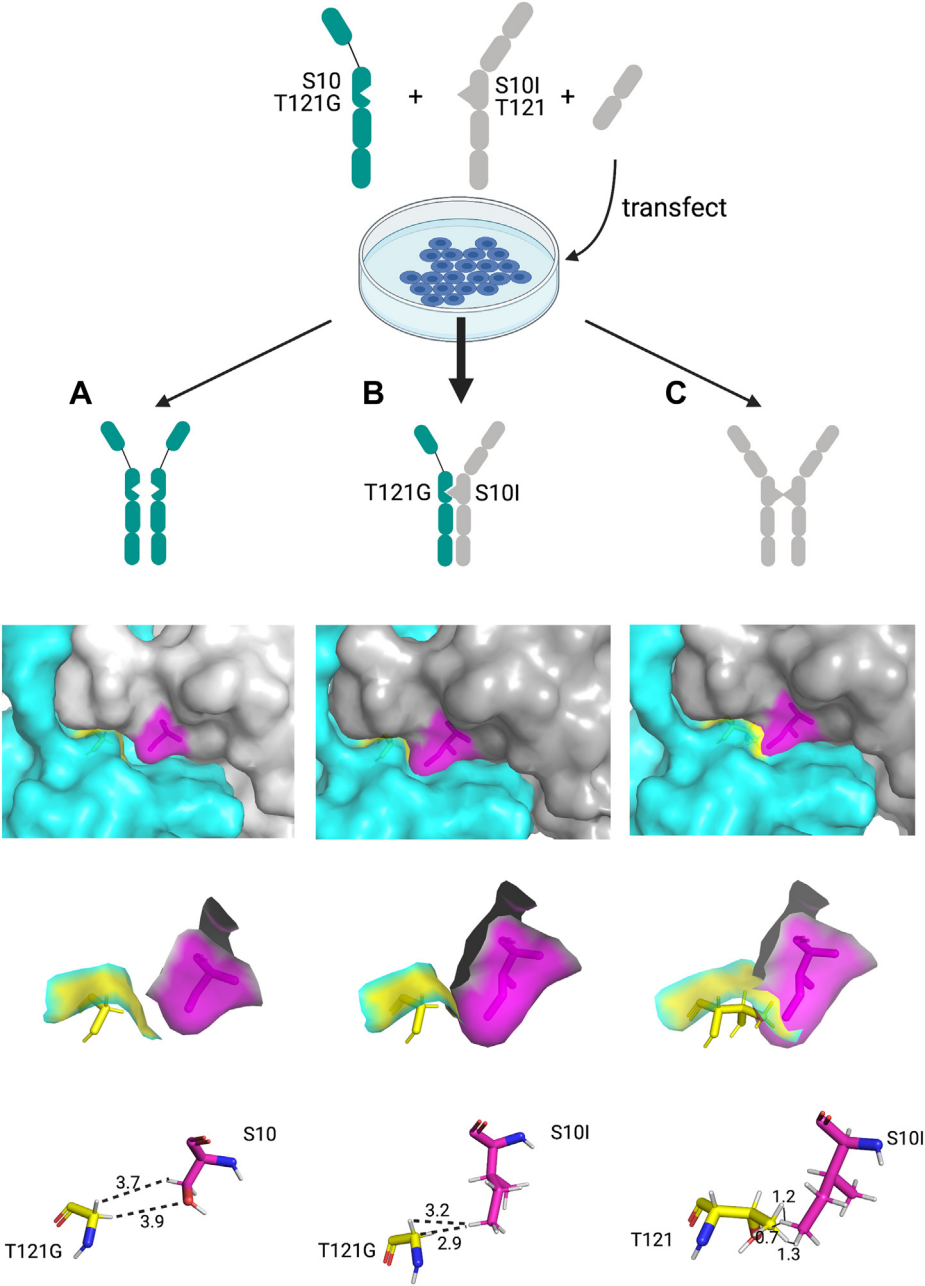
***In silico* 3D modeling of potential pairings of IgE heavy chains featuring KiH mutations.***A*, αEGFR T121G homodimer. *B*, αEGFR T121G x αPSMA S10I heterodimer. *C*, αPSMA S10I homodimer. Two heavy chains in *gray* and *cyan*; S10I in *magenta*, T121G in *yellow*. The *dashed lines* indicate the distance measurements in angstrom (Å). KiH, knobs-into-holes; PSMA, prostate-specific membrane antigen.

KiH is a commonly used heterodimerization technology for IgG molecules (21, 22). Amino acids of constant heavy domains are engineered to form a knob in one heavy chain represented by a bulky amino acid, and a hole in an opposite heavy chain represented by a small amino acid. These mutations force the heterodimerization and formation of a bsAb. In human IgE molecules, the second heavy chain domain (CH2) is responsible for the dimerization of heavy chains and is, therefore, a target for KiH engineering. The use of human CH2 IgE domains with KiH mutations has been previously reported in an EFab Domain Substitution technology (23). The authors replaced CL-CH1 domains of one arm of a bispecific IgG molecule with CH2 IgE domains featuring KiH mutations in order to prevent light chain–heavy chain mispairing (Fig. 1*A*). The mutations described are S10I in one CH2 domain, with isoleucine forming a hydrophobic knob, and T121G in an opposite CH2 domain, with glycine forming a hole. Here, we used the reported mutations in a whole IgE molecule to obtain a human bispecific IgE antibody (Fig. 1*B*). S10I point mutation was introduced in the PSMA arm, whereas T121G point mutation was introduced in the EGFR arm*. In silico* 3D modeling was used to assess the outcome of the potential interactions between the heavy chains carrying the mutations (Fig. 2). Once the expression vectors are transfected into cells, several different dimerizations could theoretically occur: EGFR T121G homodimer, EGFR T121G x PSMA S10I heterodimer, and PSMA S10I homodimer. For the EGFR T121G homodimer, the lack of the ‘knob’ mutation showed a gap in the interface between the CH2 domains (Ser10-Gly121) in the 3D model (Fig. 2*A*). At the same time, a lack of the ‘hole’ mutation in the PSMA S10I homodimer results in the multiple clashings between the two chains with the distance less than 1.5 Å (Ile10-Thr121) (Fig. 2*C*). Only the EGFR T121G x PSMA S10I heterodimer model showed an excellent KiH fit (Ile10-Gly121) (Fig. 2*B*). Therefore, the 3D modeling suggested that the EGFR T121G x PSMA S10I heterodimer would preferentially be formed over the EGFR T121G homodimer and PSMA S10I homodimer.

In a parallel approach, we introduced fos and jun peptide-based LZ pairs on the C terminus of the two different heavy chains. Fos and jun form a LZ complex (24), which we hypothesized would drive the heterodimerization of the two heavy chains (Fig. 1*C*).

**Figure 3:**
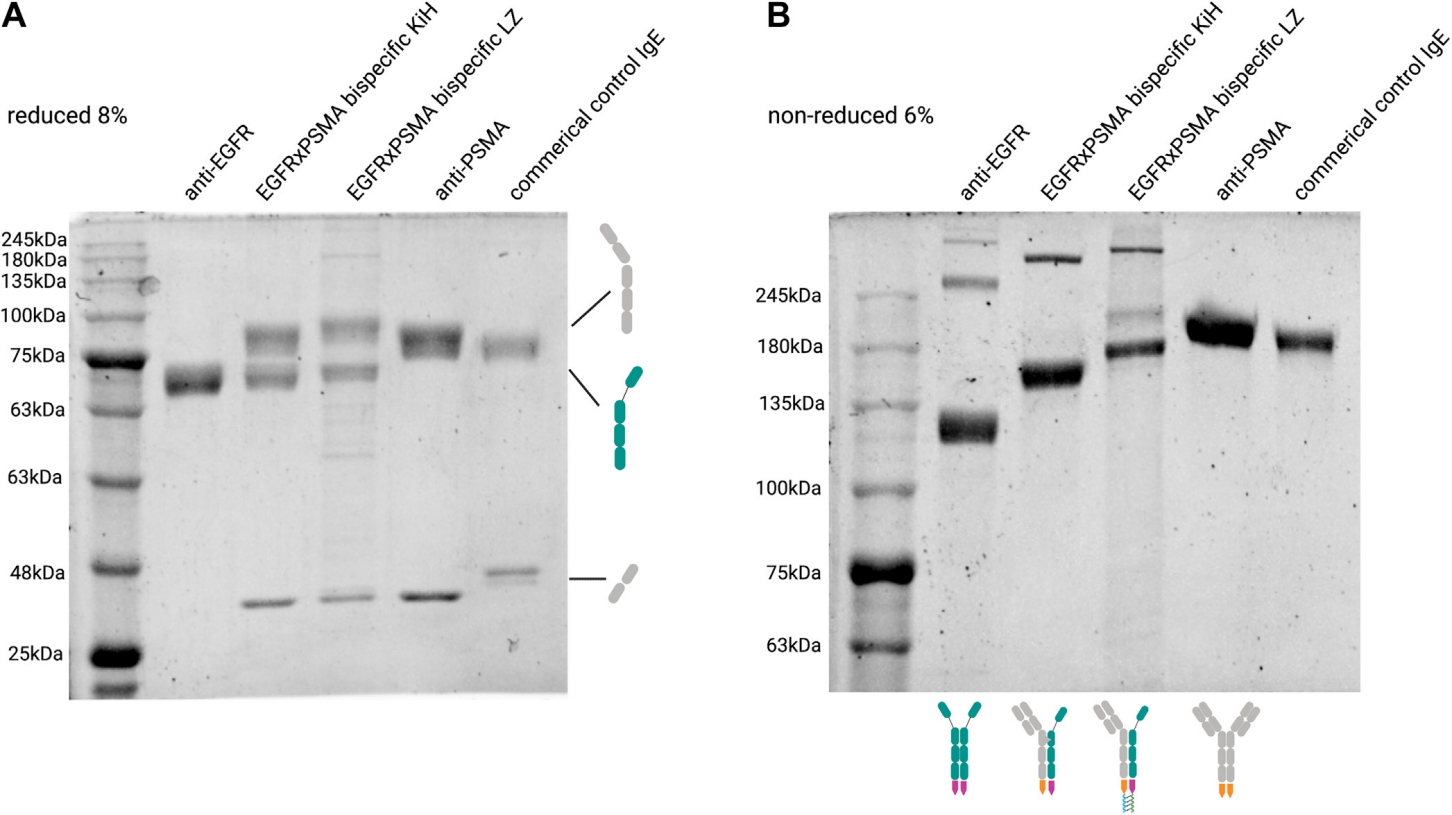
**SDS-PAGE analysis of purified IgE monospecific and bispecific antibodiesshows correct formation of bispecific IgE antibodies.** The purified antibodies were analyzed under (*A*) reducing and (*B*) nonreducing conditions.

All IgE bsAb constructs and monospecific controls were recombinantly produced. Following expression, heterocomplexes and anti-EGFR monospecific antibody were affinity purified based on the His-tag present in the EGFR arm, while anti-PSMA IgE was affinity purified using a Strep-Tactin column. The purified antibody complexes were then analyzed by SDS-PAGE to check for bispecific IgE formation (Fig. 3). As expected, under reducing conditions, one band at ∼70 kDa corresponding to the heavy chain was detected for monospecific anti-EGFR antibody; two bands at ∼90 kDa and ∼30 kDa, corresponding to heavy and light chain, respectively, were detected for monospecific anti-PSMA antibody; and three bands at ∼90 kDa, ∼70 kDa, and ∼30 kDa corresponding to anti-PSMA heavy chain, anti-EGFR heavy chain, and anti-PSMA light chain, respectively, were detected for bispecific anti-PSMAxEGFR antibodies obtained by both KiH and LZ strategies. Under nonreducing conditions, expected differences in size were detected, with monospecific anti-EGFR IgE being the smallest (∼130 kDa), followed by bispecific IgE antibody (∼170 kDa), and finally, monospecific anti-PSMA IgE (∼210 kDa). Of note, some aggregates consistently formed during purification of anti-EGFR parental antibodies and, to less extent, with bispecifics (Fig. 3*B*). The aggregate formation can be assumed to be induced by the presence of imidazole in the elution buffer (25), as we usually do not experience such aggregates with this antibody and as the anti-PSMA parental antibody purified by Strep-tag affinity chromatography was free of aggregates. Therefore, for future bsAb purifications, a His-tag independent approach might want to be considered. Furthermore, in one batch, an additional band at ∼220 kDa was observed for the LZ-containing bispecific IgE. As this band was not present in other production batches (data not shown), we assume it to be a batch-specific impurity. Nevertheless, taken together, our SDS-PAGE–based analysis of purified antibodies clearly indicated the successful formation of the bispecific IgE antibody by both KiH and LZ strategies.

To evaluate the efficacy of bispecific IgE formation, we checked for the presence of bands derived from potential unwanted pairings. After the first bispecific IgE purification step based on His-tag present in the EGFR arm, no EGFR homodimer was observed on SDS-PAGE. Next, the supernatant went through a second purification step in a Strep-tag column in order to recover the potentially formed PSMA homodimer. Also, after this second step, we detected no additional products (data not shown), indicating that the bispecific IgE complexes were formed with a high-efficiency rate, as predicted by 3D modeling (Fig. 2).

### Binding activity of bispecific IgE

**Figure 4:**
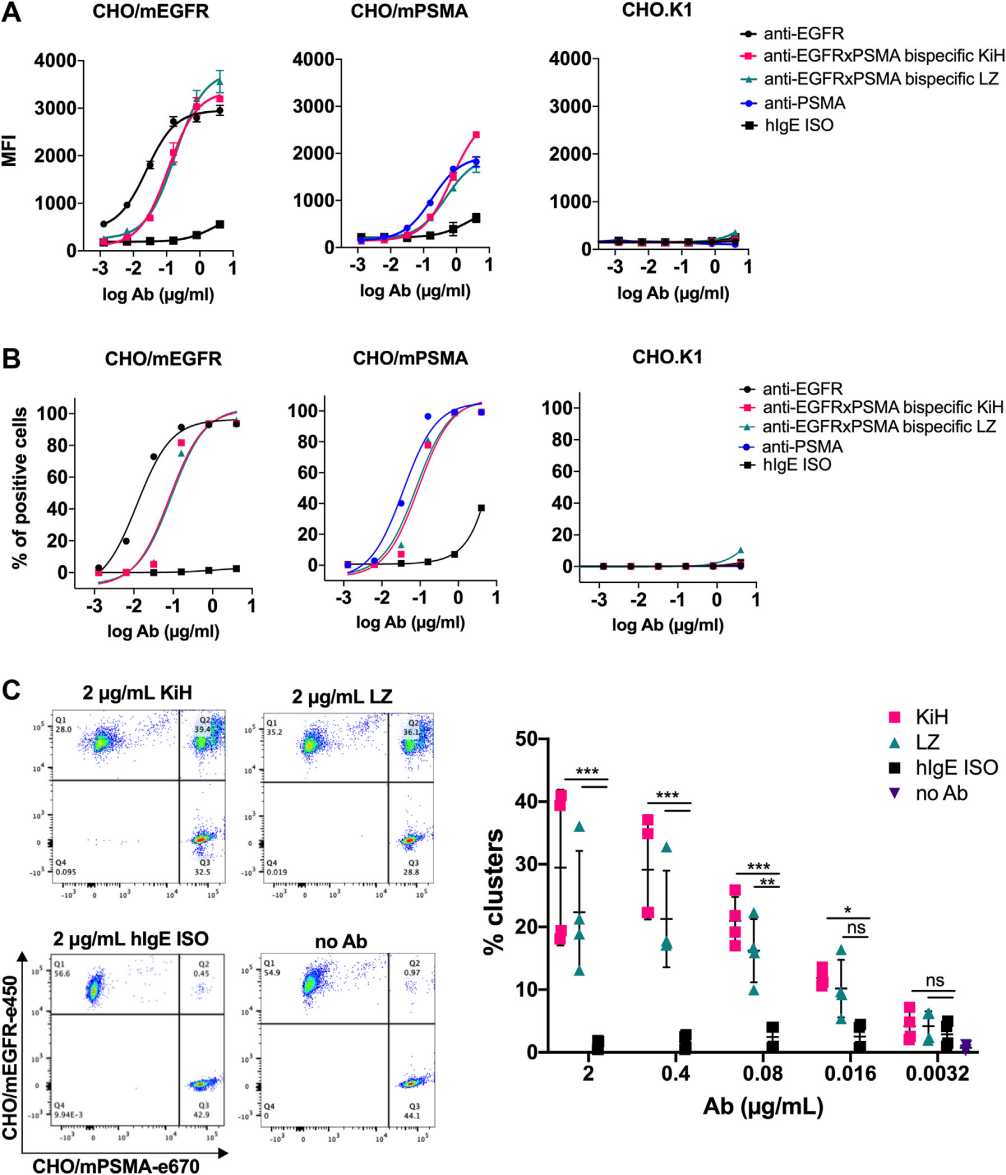
**Flow cytometry evaluation of antigen-binding properties of bispecific IgE antibodies.** Single-arm binding to CHO/mEGFR, CHO/mPSMA, and CHO.K1 cells in a dose-dependent manner represented by (*A*) MFI values and (*B*) percentage of positive cells. Each data point is the mean ± SD of duplicates. *C*, bsAbs-induced cellular clustering by simultaneous binding to CHO/mEGFR and CHO/mPSMA cells. Pooled data from two independent experiments performed in duplicates are shown. Statistical significance was determined with two-way ANOVA with Dunnett’s multiple comparison test (∗∗∗*p* < 0.0001, ∗∗*p* = 0.002, ∗*p* = 0.04). CHO, Chinese hamster ovary; MFI, mean fluorescence intensity; PSMA, prostate-specific membrane antigen.

Next, we wanted to confirm that the formed bsAbs retained their antigen-binding properties. To this end, single-arm binding of the produced anti-EGFRxPSMA bispecific IgE antibody was tested using stably transfected Chinese hamster ovary (CHO)/mPSMA and CHO/mEGFR cell lines by flow cytometry. Respective monospecific parental antibodies were used as a positive control. The monovalent-binding activity of anti-EGFRxPSMA bispecific KiH and LZ IgE antibodies to both CHO/mEGFR cells and CHO/mPSMA cells was comparable to the bivalent binding of monospecific anti-EGFR and anti-PSMA IgE antibodies, respectively (Fig. 4, *A* and *B* and Table 1). The binding of bsAb to both antigens was dose dependent. As a negative control, we used CHO.K1 cells and did not observe any binding, as expected. These results show that the specificity of both arms within the bispecific IgE antibody is preserved.

**Table 1:** EC_50_ values of tested antibodies (cell-binding assay)

EC_50_ (μg/ml)	aEGFR IgE	Bispecific KiH	Bispecific LZ	aPSMA IgE
CHO/mEGFR	0.025	0.11	0.18	/
CHO/mPSMA	/	0.73	0.48	0.18

Next, the simultaneous binding to both target antigens was tested in a cell-bridging experiment. In short, e670-labeled CHO/mPSMA cells were incubated with anti-EGFRxPSMA bispecific IgE antibodies, after which the unbound antibody was washed and e450-labeled CHO/mEGFR cells were added. Double-stained cell clusters were detected by flow cytometry, typically reaching levels of a quarter to a third of all cells measured (Fig. 4*C*).

In summary, these data show that the bispecific IgE antibody retained the binding activity toward its target antigens and its capacity for simultaneous binding of both antigens.

### The functionality of the Fc tail is preserved in the bispecific IgE molecule

In order to confirm that the introduced KiH mutations or LZ do not affect the FcεR binding, the ability to induce MC degranulation was tested. To this end, human MCs were coated with IgE antibodies, after which the crosslinking was induced with anti-IgE antibody, and degranulation was estimated as the percentage of β-hexosaminidase (β-hex) release. Anti-EGFRxPSMA bispecific IgE induced a similar level of dose-dependent MC degranulation as monospecific anti-EGFR and anti-PSMA IgE controls (Fig. S3). These results indicate that the binding of the bispecific IgE antibody to FcεR was retained.

**Figure 5:**
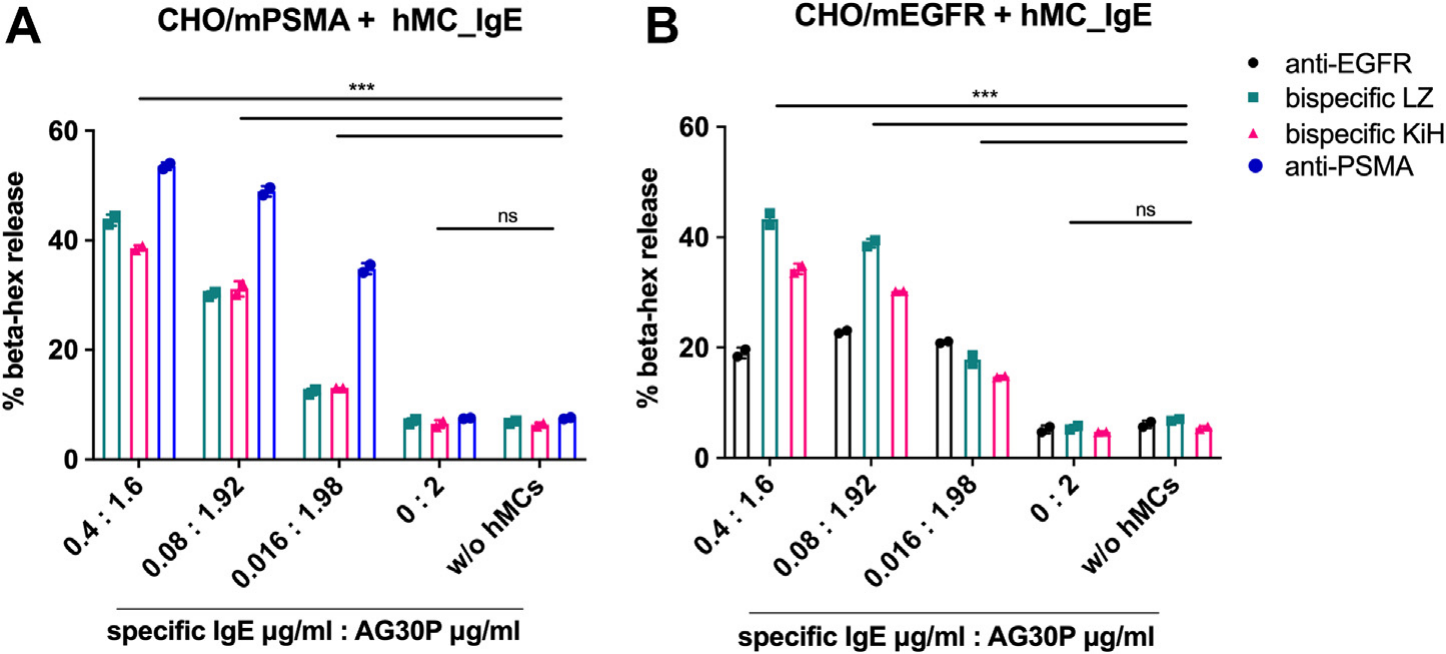
**β-hexosaminidase release after mast cell degranulation induced by bispecific IgE antibodies.** Human mast cells were sensitized with indicated mixtures of specific IgEs and AG30P unrelated IgE and added to plated (*A*) CHO/mPSMA cells or (*B*) CHO/mEGFR cells. Mean ± SD of duplicates is shown. Statistical significance was determined with two-way ANOVA with Dunnett’s multiple comparison test (*p* < 0.0001). CHO, Chinese hamster ovary; PSMA, prostate-specific membrane antigen.

Furthermore, we tested whether the bispecific IgE induces MC degranulation once it encounters the antigen-expressing cells. In this setup, human MCs coated with IgE were added to plated CHO/mPSMA or CHO/mEGFR cells, and β-hex release was measured. To show a dose-dependent effect, antigen-specific IgEs were mixed with unspecific commercial IgE (AG30P) in different ratios but maintaining the total MC coating concentration at 2 μg/ml. Bispecific IgE KiH and LZ molecules showed dose-dependent MC degranulation, reaching the highest levels of ∼40% to 50% at 0.4 μg/ml (Fig. 5, *A* and *B*). Results of the β-hex release were mirrored by IL-8 levels measured in the supernatant (Fig. S4), further confirming MC activation. Of note, anti-mPSMA IgE induced a somewhat higher level of MC degranulation upon binding to CHO/mPSMA cells (Fig. 5*A*). This could be expected as bispecific IgE molecules bind to PSMA with a single arm and, therefore, would be less efficient in inducing FcεR crosslinking that is required for MC degranulation. On the other hand, anti-EGFR IgE mediated MC degranulation was considerably lower (∼20%) in all tested conditions (Fig. 5, *A* and *B*). When anti-EGFR IgE sensitized MC was degranulated with TX100 as a positive control, the β-hexosaminidase release was also 50% lower when compared to other antibodies (data not shown). Aggregates present in the anti-EGFR IgE may likely have induced such effects (Fig. 3*B*). We observed elongated MC after the sensitization step with this IgE, indicating that the aggregates directly activated a part of MC (Fig. S5). Thus, during the sensitization, some level of activation may already have occurred, making the activated part of MC unresponsive to the crosslinking in the following step and resulting in an overall lower degranulation extent.

In summary, bispecific IgE antibodies obtained by both KiH and LZ technologies successfully induced MC degranulation upon antigen binding, indicating that both Fc-tails are functional.

### Bispecific IgE mediates cytotoxicity *in vitro* and *in vivo*

**Figure 6:**
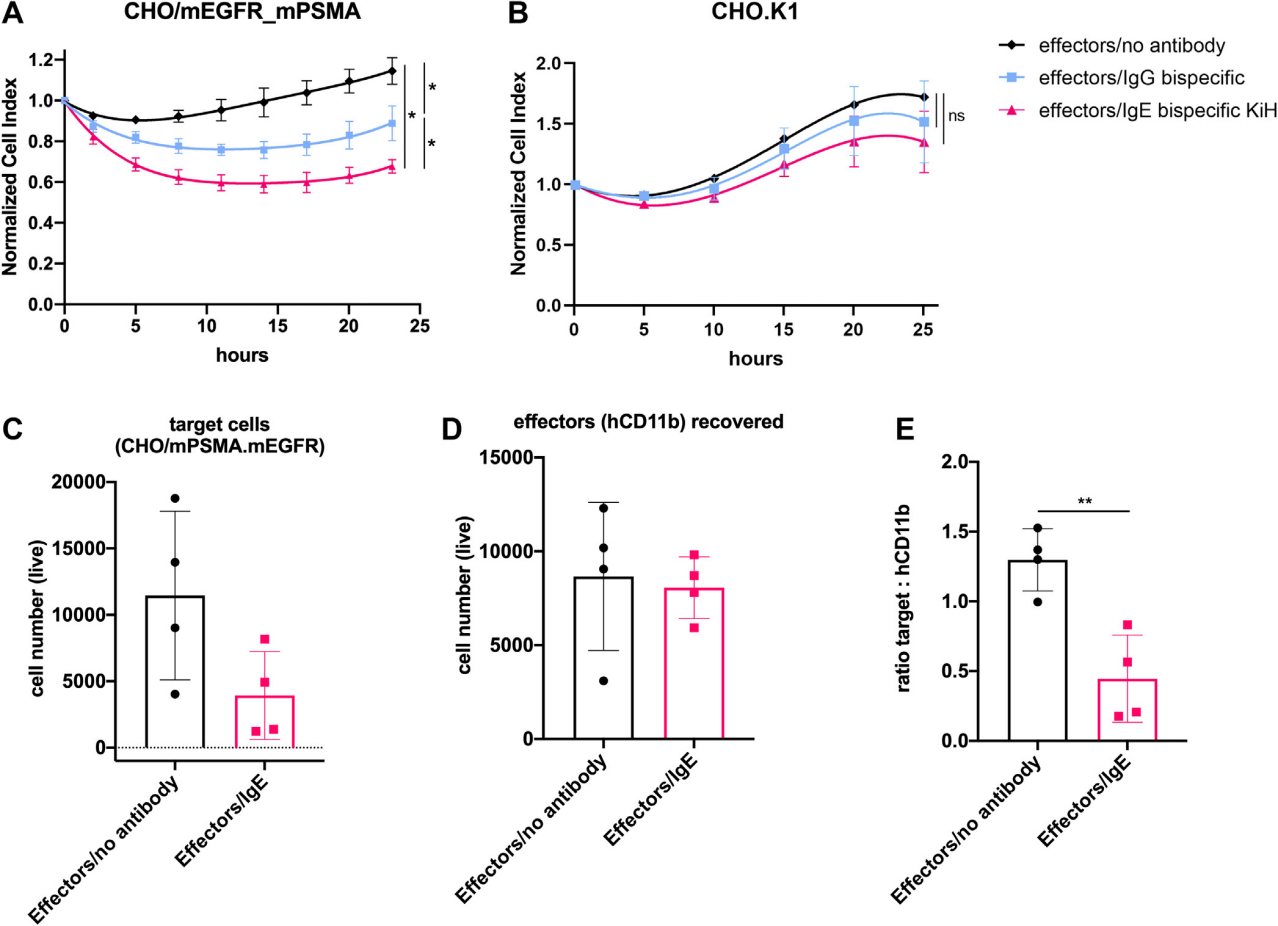
**Bispecific IgE induces cytotoxicity *in vitro* and *in vivo*.** Real-time ADCC assay against (*A*) CHO/mPSMA.mEGFR cells and (*B*) CHO.K1 cells and hCD11b+ cells as effectors. Representative data of two independent experiments performed in duplicates are shown. Normalization was done ∼5 h after the effector cell addition. Statistical significance was determined using an extra sum-of-squares F test (*p* < 0.0001) (*C*) Rag2^−/−^γc^−/−^ mice were intraperitoneally injected with CMFDA-labeled CHO/mPSMA.mEGFR target cells, indicated antibodies and hCD11b+ cells as effectors. Recovered number of live target cells (*C*) and hCD11b+ cells (*D*) after 24 h is shown. *E*, ratio between recovered number of live target cells and hCD11b+ cells after 24 h. Statistical significance was determined by unpaired two-tailed *t* test (*p* = 0.0044). (n = 4). CHO, Chinese hamster ovary; PSMA, prostate-specific membrane antigen.

Next, to fully confirm the functionality of IgE bsAb, we tested whether it is capable of inducing cytotoxicity. Thus, we performed *in vitro* ADCC assays targeting CHO/mPSMA_mEGFR cells, using purified human peripheral blood CD11b^+^ cells as effector cells. In this set of experiments, we directly compared IgE bsAb made by KiH strategy to IgG1 bsAb with the same specificity. Our data show that IgE bsAb induced cell death at a range of ∼40%, which was consistently significantly higher than IgG1 bsAb, inducing around 25% cell death (Fig. 6*A*). No unspecific ADCC effect was observed against CHO.K1 cells used as a control (Fig. 6*B*). The level of aggregates present in bispecific IgE samples was not high enough to induce MC activation in the MC degranulation experiments (Fig. 5). Therefore, it is highly unlikely that the low amount of aggregates present in IgE bsAb preparations may have caused any biological effect in the ADCC experiments. Such an assumption is further supported by the absence of killing of CHO.K1 cells (Fig. 6*B*). Thus, taken together, our data demonstrate that IgE bsAb-mediated ADCC is more efficient than that of its IgG1 bsAb homolog, indicating a greater cytotoxic potential of IgE isotype when CD11b+ cells are provided as effector cells.

Finally, we studied the antibody-mediated *in vivo* cell killing in a murine model. To this end, Rag2^−/−^γc^−/−^ immunodeficient mice were injected with 5-chloromethylfluorescein diacetate (CMFDA)–labeled CHO/mPSMA_EGFR cells, human CD11b^+^ effector cells, and bispecific IgE. As human IgG1 is crossreactive with murine Fcγ receptors and has been reported to induce tumor cell killing by activating murine effector cells (26), we omitted a direct control of IgG-mediated *in vivo* killing. After 24 h, transferred cells were recovered by peritoneal lavage, and the number of remaining target cells (CHO/mPSMA_EGFR) and CD11b^+^ cells were assessed by flow cytometry. The number of recovered live target cells was lower in IgE-treated mice when compared to the control group (Fig. 6*C*). In contrast, the recovered CD11b+ effector cell population remained the same in the mouse groups that received IgE bsAb or not (Fig. 6*D*). In order to internally normalize the number of recovered cells, we used the ratio between the numbers of live target cells and live CD11b^+^ effector cells. Tumour/effector ratio was significantly lower in IgE-treated mice when compared to the control group (Fig. 6*E*), clearly demonstrating IgE induced the cell killing *in vivo*.

In conclusion, IgE bsAb exhibited greater *in vitro* cytotoxic capacity than its IgG counterpart and was able to mediate *in vivo* target cell killing.

## Discussion

The introduction of mAbs in clinical settings fundamentally changed the current way cancer is treated, achieving success in previously untreatable types of cancer. Furthermore, the most recent clinical approval of amivantamab (6), a bsAb targeting the EGFR and cMet, has made apparent that the dual TAAs targeting bsAbs can show improved efficacy over monotherapy.

However, ever since it became evident that the Fc part plays an important role in the antibody efficacy, a tremendous effort has been made to further optimize IgG molecules, the commonly used antibody isotype. The Fc engineering focused on various aspects, including: increasing the half-life by improving the binding to FcRn, optimizing the activation *versus* inhibition (A/I) ratio by reducing the interaction of the Fc-part with inhibitory FcγRIIb and increasing the binding to activatory FcγRs, and optimizing complement activation (7). Switching to the IgE isotype solves two of the mentioned aspects. IgE is characterized by extended tissue half-life and lacks binding to inhibitory Fc receptors. In addition, IgE has been shown to offer superior macrophage tumor infiltration and the ability to re-educate them toward the M1 phenotype, which resulted in better tumor control when compared to IgG (8). Given the promising role of IgE-based cancer therapeutics, the bispecific IgE antibody represents a very attractive novel antibody format that would combine the advantages of IgE isotype with increased tumor specificity characteristic for bispecific molecules. The work presented here describes the successful generation of IgE bsAbs by two different approaches—KiH and LZ-mediated pairing.

We made use of the currently still unconventional Fab×sdAb-Fc format in both strategies (19). This format allowed us to clearly demonstrate by SDS-PAGE analysis that IgE bsAb formation by both KiH and LZ approaches was highly successful and that unwanted pairings were absent. These findings suggest that described strategies efficiently drive IgE heavy chain heterodimerization. In order to avoid light and heavy chain mismatching in more conventional bsAb formats, where two heavy chains and two light chains are present, some alternative solutions have been described for IgG-based bsAb, which could be adapted for bispecific IgE molecules as well (27, 28).

Our data show that the bispecific IgE antibodies preserved antigen-binding properties and antigen specificity of both arms in addition to the fully functional Fc part, as they induced MC degranulation upon antigen binding. An important finding of this study is the superior ADCC effect of bispecific IgE compared to its IgG bispecific homolog with the exact variable domains and, hence, identical antigen-binding properties. Here, human CD11b^+^ cells, mainly represented by monocytes, were provided as effector cells, further supporting previously published findings of superior macrophage activation by IgE isotype, including the subsets likely found in the tumor microenvironment (8, 11, 29). Macrophages are the most abundant type of immune cells in the tumor environment (30). Thus, our findings strongly suggest that IgE isotype-based antibodies, including bispecifics, can be therapeutically superior to those with an IgG Fc-part.

As IgE antibodies not only bind to macrophages but also to MCs and basophils and are well known to induce allergic reactions, the aspect of anaphylaxis has been a great concern for the use of IgE mAbs in tumor therapy. However, preclinical (31, 32, 33) and initial clinical safety data (12) with tumor antigens targeting monospecific IgE antibodies did not suggest any increased risks, and the safety profile appeared satisfactory. Systemic allergic reactions can be a risk if IgE antibodies encounter circulating tumor cells expressing the target antigen. As circulating tumor cells are generally present in only minute quantities, such a reaction appears highly unlikely for both monospecific and bispecific IgE antibodies. Furthermore, as bsAbs can substantially increase target selectivity, we would like to argue that the bispecific IgE format may even further improve the safety of IgE therapy and reduce the risk of systemic off-site reactions.

Furthermore, as with all proof-of-concept studies, our work also has certain limitations. For instance, the antigens and model systems chosen for this study were somewhat artificial. However, the described technology for IgE bispecific generation can now be used to target physiologically more relevant tumor antigens that would allow us to test the *in vivo* effect in immunocompetent human IgεR-transgenic preclinical models and, consecutively, in a clinical setting.

In conclusion, we report the generation of fully functional tumor targeting human IgE bsAb with preserved antigen binding and superior cytotoxic capacity than its IgG homolog. The described IgE bispecific formats open a wide area for further IgE exploitation as cancer therapeutics by arming the potent IgE molecules with increased tumor specificity.

## Experimental procedures

### Cell culture

HEK293T cells were grown in Iscove's modified Dulbecco's medium (Gibco) supplemented with 10% heat-inactivated fetal bovine serum (FBS) (Gibco), 1% penicillin/streptomycin (Gibco), 2 mM L-glutamine (Gibco), and 50 μM 2-mercaptoethanol (Gibco). CHO.K1 cells (ATCC; catalog no.: # CCL-61) were grown in Advanced Dulbecco's modified Eagle’s medium/Ham's F-12, (DMEM/F-12; Gibco), supplemented with 5% heat-inactivated FBS, 2 mM L-glutamine, and 1% penicillin/streptomycin. Culture conditions were maintained at 37 °C in a humidified atmosphere containing 7% CO_2_.

FreeStyle 293 cells (Invitrogen) were grown in FreeStyle 293 Expression medium (Invitrogen) on a CO_2_ resistant shaker (Thermo Scientific) at 37 °C, 8% CO_2_, and 120 rpm.

CHO/mPSMA and CHO/mEGFR stable transfected cell lines were previously developed within our research group (19). They were grown in Advanced DMEM/F12 medium supplemented with 5% heat-inactivated FBS, 1% penicillin/streptomycin, 2 mM L-glutamine, and 0.8 mg/ml G418 (Gibco). Culture conditions were maintained at 37 °C in a humidified atmosphere containing 7% CO_2_.

CHO/mEGFR_mPSMA cell line was obtained by stably transfecting CHO/mEGFR with pcDNA3.1/hygro+.mPSMA as described previously (19). The stable clone was selected after 10 to 14 days of growth under hygromycin (0.6 mg/ml) and geneticin (0.8 mg/ml) pressure, after which single-cell sorting was performed on a FACSMelody (BD). The expression was regularly monitored by flow cytometry with an anti-mPSMA antibody (Sam103 (19)), and the selected clone showed stable PSMA expression for more than a month.

For ADCC assay, the CHO/mEGFR_mPSMA cells were grown in DMEM/F12 (Gibco; 11320033) supplemented with 5% heat-inactivated FBS, 1% penicillin/streptomycin, 0.8 mg/ml geneticin, and 0.6 mg/ml hygromycin. Culture conditions were maintained at 37 °C in a humidified atmosphere containing 7% CO_2_.

### Design of vectors

The anti-mEGFR sdAb (RR359) has been described before (20), and the amino acid sequence of the anti-mPSMA antibody (Sam103) was obtained from patent US20170342169A1. The sequence of IgE constant heavy chain was obtained from the UniProt database (ID: P01854). The sdAb (anti-EGFR) was linked to CH2-CH4 IgE constant domains *via* a partial llama linker (EPKTPKPQPQPQPQP).

Signal peptides were introduced at the N terminus. For KiH technology, an S10I point mutation was introduced in the CH2 domain of the PSMA arm, whereas T121G point mutation was introduced in the CH2 domain of the EGFR arm. At the C terminus of PSMA and EGFR arms, Strep-tag and His-tag were introduced, respectively. The tags were followed by a stop codon, thrombin cleavage site, and fos or jun LZ sequences, respectively. An additional thrombin cleavage site was introduced upstream fos/jun sequences so that the LZ can be removed by thrombin digestion after antibody purification, if needed. However, for this set of experiments, the LZ had not been cleaved off. These initial mAb expression vectors were constructed by *de novo* synthesis (Biomatik). Some of the expression vectors (for parental monospecific antibodies and anti-EGFR-jun) were then obtained by Q5 site-directed mutagenesis in order to reverse KiH mutations and delete stop codons. The success of site-directed mutagenesis was confirmed by Sanger sequencing (Genewiz). For all antibodies, pcDNA3.1(+) was used as an expression vector. A schematic representation of IgE antibody constructs is given in Fig. S1. Bispecific human IgG1 featured the same variable chains as IgE. It was obtained by previously described KiH mutations (T366S, L368A, and Y407V point mutations were introduced to the CH3 region of the EGFR arm, a T366W point mutation was introduced to the CH3 region of the PSMA arm). Amino acid sequences of all antibodies are given in Table S1.

### *In silico* modeling

*In silico* modeling was done using PyMOL Molecular Graphics System (version 2.3.3, Schrödinger, LLC). Mutant models were generated using the reported crystal structure of IgE Fc (Protein Data Bank entry 2WQR) as a starting model. Water molecules were removed, adaptive Poisson–Boltzmann solver electrostatics calculations performed, and indicated amino acids were mutated. For the mutation T121G, there are no rotamers for Glycine. For the mutation S10I, we used isoleucine rotamer with highest frequency suggested by PyMOL (60%).

### Recombinant antibody production

Bispecific IgE antibodies and anti-EGFR IgE antibody were recombinantly produced in HEK293T cells. Briefly, pcDNA3.1(+) expression vectors encoding corresponding heavy and light chains (1:1 ratio for monospecific antibody, 1:1:1 ratio for bsAbs) were transfected into HEK293T cells (80% confluent) using the Lipofectamine 2000 reagent (Invitrogen) according to the manufacturer’s recommendation. After 6 h, the cells were gently washed with PBS and fresh FreeStyle 293 Expression Medium (Invitrogen) was added. The cells were incubated for 7 days at 37 °C in a humidified atmosphere containing 7% CO_2_. On day 7 post-transfection, the cell suspension was collected and centrifuged for 15 min at 2500*g*. The supernatants were filtered over a 0.22 μm filter and stored at 4 °C.

Anti-PSMA IgE antibody and bispecific IgG1 antibody were recombinantly produced in FreeStyle293 cells. Briefly, the cells were transfected with pcDNA3.1(+) expression vectors encoding corresponding heavy and light chains (1:1:1 ratio for bispecific antibody), using 293fectin reagent (Invitrogen) according to the manufacturer’s recommendation. The cells were incubated for 7 days at 37 °C, with 8% CO_2_ at 120 rpm. On day 7 post-transfection, the cell suspension was collected and centrifuged for 15 min at 2500*g*. The supernatants were filtered over a 0.22 μm filter and stored at 4 °C.

### His-tag–based antibody purification

The supernatant containing the IgE antibodies was mixed with a predetermined amount of Ni Sepharose excel (Cytiva) and rotated overnight at 4 °C. Following overnight capturing, the resin was washed once with PBS, and the bound antibody was purified from the resin by two-step elution with 50 mM imidazole pH 7.5, followed by 1 M imidazole pH 7.5. The antibody was rebuffered to PBS using either Zeba Spin (Thermo Scientific) or PD-10 (Cytiva) desalting columns according to the manufacturer’s instructions.

### Strep-tag–based antibody purification

To neutralize the biotin present in the FreeStyle293 medium, which can interfere with the Strep-Tactin column, a BioLock solution (Iba LifeSciences) was added to the supernatant containing IgE antibodies according to the manufacturer’s recommendation. Next, the supernatant was loaded onto a manually packed Strep-Tactin column (CV = 2 ml) previously equilibrated in 100 mM Tris–HCl pH 8.0, 150 mM NaCl, and 1 mM EDTA. The antibody was eluted with a buffer (100 mM Tris–HCl, pH 8.0150 mM NaCl, and 1 mM EDTA) containing 2.5 mM desthiobiotin into 1 ml fractions. The fractions were analyzed by SDS-PAGE, and the ones that contained the antibody were combined. The antibody was rebuffered to PBS using either Zeba Spin or PD-10 desalting columns according to the manufacturer’s instructions.

### IgG purification

Bispecific IgG antibody was purified as described before (19) by protein A affinity chromatography.

### Antibody quantification

All antibodies were quantified using a NanoDrop spectrophotometer at 280 nm. Antibody concentration was calculated based on the Beer–Lambert Law, A = ε ∗ b ∗ c, (A is the *A*_280_ absorbance, b is the path length, c is the analyte concentration, and ε is the wavelength-dependent molar absorptivity coefficient with units of M^−1^ cm^−1^). The coefficients were calculated using the ProtParam online tool based on the amino acid sequences.

### SDS-PAGE

One microgram of each antibody was diluted in Laemmli sample buffer (Bio-Rad) for analysis under nonreducing conditions or in Laemmli sample buffer containing 10% ß-mercaptoethanol for analysis under reducing conditions. Samples were heated either at 95 °C for 5 min (reduced) or at 90 °C for 2 min (nonreduced). Samples and BLUeye Prestained Protein Ladder (Geneflow) were loaded to either 8% polyacrylamide gel (reduced samples) or 6% polyacrylamide gel (nonreduced samples). The gels were run at 70 V for 30 min, after which the voltage was increased to 120 V for 60 min. Gels were stained using GelCode Blue Safe Protein Stain (Thermo Scientific), fixed for 30 min in buffer containing 40% ethanol and 10% acetic acid, destained with distilled water, and scanned using ChemiDocTM Touch Imaging System (Bio-Rad).

### Cell-binding assays by flow cytometry

CHO.K1, CHO/mEGFR, and CHO/mPSMA cells were detached and washed once with flow cytometry staining buffer (FACS buffer: PBS + 2% FBS). Indicated antibodies were added in fivefold serial dilution in FACS buffer and incubated at 4 °C for 45 min. Secondary antibody staining was done with goat antihuman IgE antibody conjugated with FITC (Novus Bio) in 1:1000 dilution in FACS buffer at 4 °C for 45 min. Samples were analyzed by FACS CantoTM II (BD) using the software program BD FACSDiva (BD Biosciences). EC50 values were calculated in GraphPad Prism (GraphPad Software).

To test the simultaneous antigen-binding activity of anti-PSMA x EGFR bsAb, the cell bridging experiment was used as described previously (19) with some modifications. To prepare a single-cell suspension, CHO/mPSMA and CHO/mEGFR cells were detached from flasks and washed twice with PBS. CHO/mPSMA and CHO/mEGFR cells were labeled with cell staining dye eFluor 450 or 670 (eBioscience), respectively, according to the manufacturer’s recommendations. About 5 × 10^4^ labeled CHO/mPSMA cells were mixed in 50 μl FACS buffer and incubated with indicated concentrations of anti-PSMA x EGFR bsAb at 4 °C for 45 min. The cells were washed three times with FACS buffer and 5 × 10^4^ labeled CHO/mEGFR cells were added. The cells were incubated at 4 °C for 45 min and analyzed on a FACS CantoTM II (BD) using the software program BD FACSDiva.

### Human MC generation

Human peripheral blood mononuclear cell (PBMC)–derived MCs were generated from buffy coats as previously described by Folkerts *et al.* (34) Buffy coats were obtained from healthy donors (Sanquin). Before sample collection, written consent was obtained. In short, buffy coats were used to obtain PBMCs, after which CD34^+^ precursor cells were isolated using the EasySep Human CD34 Positive Selection Kit (STEMCELL Technologies). CD34^+^ cells were cultured for 4 weeks under serum-free conditions in StemSpan medium (STEMCELL Technologies) supplemented with recombinant human IL-6 (50 ng/ml; Peprotech), human IL-3 (10 ng/ml; Peprotech), and human Stem Cell Factor (100 ng/ml PeproTech). After 4 weeks, the cell culture medium was changed to Iscove's modified Dulbecco's medium supplemented with 0.5% bovine serum albumin, human IL-6 (50 ng/ml, PeproTech), and 3% supernatant of CHO transfectants secreting murine stem cell factor (a gift from Dr P. Dubreuil). The mature MCs were identified based on the expression of CD117 (eBioscience) and FcεRIa (eBioscience) by flow cytometry using BD FACS Canto II (approximately 90%).

### Human MC degranulation assay (β-hex assay and IL8 ELISA)

CHO/mEGFR and CHO/mPSMA cells were plated in 96-well plates at the density of 10,000 and 15,000 cells/well, respectively, and cultured for 2 days to ∼80% confluency. Human PBMC-derived MCs were primed with the indicated concentrations of IgE antibodies at 37 °C overnight, after which the unbound IgE was washed, and sensitized MCs were either added to plated CHO cells washed with PBS (20,000 MCs/well) or the crosslinking was induced with 1.25 to 10 μg/ml antihuman IgE antibody (Dako; #A0094). Myeloma IgE clone AG30P (Merck Millipore) was used as a negative control. After 2 h, supernatants were collected and either used immediately in the β-hex assay or the aliquots were frozen for IL-8 quantification.

For β-hex assay, the supernatants were mixed with 200 μM 4-methylumbelliferyl-β-d-glucosaminide substrate in 100 mM citric acid, pH 4.5, for 1 h at 37 °C. The enzymatic reaction was stopped with 0.1 M glycine buffer, pH 10.7. As a positive control, cells were lysed with 0.2% Triton X-100, in order to determine total β-hex content. The β-hex content was quantified by fluorometric measurements at ex360/em452 nm. The percentage of released β-hex was calculated by using the following equation: $\frac{A-B}{T-B}\;x100\%$, where *A* is the amount of β-hex released from stimulated cells, *B* is the amount of β-hex released from unstimulated cells, and *T* is total β-hex content.

IL-8 was quantified with the IL-8 ELISA kit (Invitrogen; #88-8086-88), as per the manufacturer’s recommendations.

### Human subjects and sample collection for ADCC

This study was approved by the Clinica Universidad de Navarra Ethics Committee (2019.139) and Navarra Blood and Tissue Bank Navarrabiomed Biobank. We have complied with all relevant ethical regulations for working with human participants in accordance with the Declaration of Helsinki.

### Isolation of human CD11b^+^ cells from donor blood

To isolate PBMCs, the donor blood was submitted to Ficoll-Paque (Cytiva; 17144003) gradient centrifugation, after which the buffy coat was collected, washed with PBS, and submitted to red blood cell lysis with ammonium–chloride–potassium lysing buffer (Gibco; A1049201). After stopping the lysis reaction, the PBMCs were passed through a 70 μm cell strainer and washed with PBS. Next, PBMCs were used to isolate CD11b^+^ cells using CD11b MicroBeads (Miltenyi Biotec; 130-049-601) as per the manufacturer’s protocol.

### ADCC assay

The ADCC assay was performed in xCELLigence Real-Time Cell Analysis Instrument (Agilent, xCELLigence RTCA DP), an impedance-based technology that monitors cell proliferation and attachment in real-time. The day before starting the assay, target CHO/mPSMA_mEGFR cells were plated in E-Plate 16 PET (Agilent #300600890), 40,000 cells/well, to allow them to adhere and reach the exponential growth phase. The following day the human CD11b^+^ effector cells were preincubated with indicated IgE/IgG antibodies at 5 μg/ml for 1 h, at 37 °C and 5% CO_2_, after which the unbound antibodies were washed, and 80,000 of IgE- or IgG-coated effector cells were added per well. The cell killing was monitored by the instrument.

### *In vivo* cytotoxicity assay

BALB/c.Rag2^tmlFwa^ IL2rg^tmlWjl^ (Rag2^−/−^γc^−/−^) mice were bred and maintained in the animal facility of Centro de investigación médica aplicada (CIMA), Universidad de Navarra maintained at CIMA under the guidelines of the Ethics Committee of the center. CHO/mEGFR_mPSMA cells were detached and prestained with CellTracker Green CMFDA Dye (ThermoFisher Scientific; #C7025, 1:1500 dilution) according to manufacturer’s protocol for cells in suspension. The mice were intraperitoneally injected with 1.5 × 10^6^ CMFDA-prestained CHO/mEGFR_mPSMA cells into the left side of the peritoneal cavity. Straight after, they received a separate injection into the right side of the peritoneal cavity containing 3 × 10^6^ human CD11b^+^ cells isolated from a fresh buffy coat as described previously, together with 50 μg of indicated bispecific antibodies. After 24 h, we performed the peritoneal lavage with 3 ml of PBS, and the cell populations were quantified by flow cytometry on CytoFLEX S (BD; B75408). The staining included antimouse CD45.2 (Biolegend #109820, 1:200) in order to exclude murine leukocytes, antihuman CD11b PerCP/Cy5.5 (Biolegend #301328, 1:100) for effector population detection, and Zombie NIR (Biolegend #423105, 1:1000) for live/dead. The gating strategy is shown in Fig. S2.

### Statistical analysis

GraphPad Prism V.8.2.1 software (GraphPad Software) was used for statistical analysis. Mean differences were compared using *t* tests (for comparisons of two groups) or two-way ANOVA tests (for comparisons of three or more groups). Longitudinal data were fitted to a fourth-order polynomial equation and compared with an extra sum-of-squares F test. Values of *p* < 0.05 were considered to be statistically significant.

## Data availability

All data generated in this study are available within the article and its supplementary data files.

## Supporting information

This article contains supporting information.

## Conflict of interest

The authors declare that they have no conflicts of interest with the contents of this article.
