# Supplementary material for: A human IgE bispecific antibody shows potent cytotoxic capacity mediated by monocytes
Source: J Biol Chem. 2022 Jun 16;298(8):102153. doi: 10.1016/j.jbc.2022.102153 (PMC9293656; doi:10.1016/j.jbc.2022.102153)
Supplement: Supplemental Figures S1–S5 and Table S1 [file mmc1.docx]

**Supplementary**

**Supplementary Table 1. Amino acid sequences of designed antibodies.**

| **SAM103(anti-mPSMA) fos IgE heavy chain -> leucine zippers bsAb** |
| --- |
| MAVLGLLFCLVTFPSCVLSEVKLVESEGGLVQPGSSMKLSCTASGFTFSDYYMAWVRQVPEKGLEWVANINYDGTTTYYLDSLKSRFIISRDNSKNILYLQMSSLKSEDTATYYCARVLDGYYGYFDYWGQGTTLSVSSASTQSPSVFPLTRCCKNIPSNATSVTLGCLATGYFPEPVMVTWDTGSLNGTTMTLPATTLTLSGHYATISLLTVSGAWAKQMFTCRVAHTPSSTDWVDNKTFSVCSRDFTPPTVKILQSSCDGGGHFPPTIQLLCLVSGYTPGTINITWLEDGQVMDVDLSTASTTQEGELASTQSELTLSQKHWLSDRTYTCQVTYQGHTFEDSTKKCADSNPRGVSAYLSRPSPFDLFIRKSPTITCLVVDLAPSKGTVNLTWSRASGKPVNHSTRKEEKQRNGTLTVTSTLPVGTRDWIEGETYQCRVTHPHLPRALMRSTTKTSGPRAAPEVYAFATPEWPGSRDKRTLACLIQNFMPEDISVQWLHNEVQLPDARHSTTQPRKTKGSGFFVFSRLEVTRAEWEQKDEFICRAVHEAASPSQTVQRAVSVNPGKGGSAWSHPQFEKGGLVPRGSGGGSGLTDTLQAETDQLEDEKSALQTEIANLLKEKEKLEFILAA |
| **SAM103(anti-mPSMA) S10I IgE heavy chain -> KiH bsAb** |
| MAVLGLLFCLVTFPSCVLSEVKLVESEGGLVQPGSSMKLSCTASGFTFSDYYMAWVRQVPEKGLEWVANINYDGTTTYYLDSLKSRFIISRDNSKNILYLQMSSLKSEDTATYYCARVLDGYYGYFDYWGQGTTLSVSSASTQSPSVFPLTRCCKNIPSNATSVTLGCLATGYFPEPVMVTWDTGSLNGTTMTLPATTLTLSGHYATISLLTVSGAWAKQMFTCRVAHTPSSTDWVDNKTFSVCSRDFTPPTVKILQS**I**CDGGGHFPPTIQLLCLVSGYTPGTINITWLEDGQVMDVDLSTASTTQEGELASTQSELTLSQKHWLSDRTYTCQVTYQGHTFEDSTKKCADSNPRGVSAYLSRPSPFDLFIRKSPTITCLVVDLAPSKGTVNLTWSRASGKPVNHSTRKEEKQRNGTLTVTSTLPVGTRDWIEGETYQCRVTHPHLPRALMRSTTKTSGPRAAPEVYAFATPEWPGSRDKRTLACLIQNFMPEDISVQWLHNEVQLPDARHSTTQPRKTKGSGFFVFSRLEVTRAEWEQKDEFICRAVHEAASPSQTVQRAVSVNPGKGGSAWSHPQFEK |
| **SAM103(anti-mPSMA) IgE heavy chain -> monospecific parental Ab** |
| MAVLGLLFCLVTFPSCVLSEVKLVESEGGLVQPGSSMKLSCTASGFTFSDYYMAWVRQVPEKGLEWVANINYDGTTTYYLDSLKSRFIISRDNSKNILYLQMSSLKSEDTATYYCARVLDGYYGYFDYWGQGTTLSVSSASTQSPSVFPLTRCCKNIPSNATSVTLGCLATGYFPEPVMVTWDTGSLNGTTMTLPATTLTLSGHYATISLLTVSGAWAKQMFTCRVAHTPSSTDWVDNKTFSVCSRDFTPPTVKILQSSCDGGGHFPPTIQLLCLVSGYTPGTINITWLEDGQVMDVDLSTASTTQEGELASTQSELTLSQKHWLSDRTYTCQVTYQGHTFEDSTKKCADSNPRGVSAYLSRPSPFDLFIRKSPTITCLVVDLAPSKGTVNLTWSRASGKPVNHSTRKEEKQRNGTLTVTSTLPVGTRDWIEGETYQCRVTHPHLPRALMRSTTKTSGPRAAPEVYAFATPEWPGSRDKRTLACLIQNFMPEDISVQWLHNEVQLPDARHSTTQPRKTKGSGFFVFSRLEVTRAEWEQKDEFICRAVHEAASPSQTVQRAVSVNPGKGGSAWSHPQFEK |
| **SAM103(anti-mPSMA)kappa light chain** |
| MSVLTQVLALLLLWLTGARCQIVLTQSPAIMSASPGEKVTISCSASSSVSYMYWYQQKPGSSPKPWIYRTYNLASGVPARFSGSGSGTSYSLTISSMEAEDAATYYCQQSHTYPPTFGGGTKLEIKRTVAAPSVFIFPPSDEQLKSGTASVVCLLNNFYPREAKVQWKVDNALQSGNSQESVTEQDSKDSTYSLSSTLTLSKADYEKHKVYACEVTHQGLSSPVTKSFNRGEC |
| **RR359(anti-mEGFR) jun IgE heavy chain -> leucine zippers bsAb** |
| MAVLGLLFCLVTFPSCVLSQVQLQESGGGLVQAGGSLRLSCAASGRTFTSYAMGWFRQVPGKEREFVAALSTRSAGNTYYADSVKGRFTISRDNAKNTVYLQMSSLKAEDTAVYYCAAGYMSSDADPSLAASLHPYDYWGQGTQVTVSSEPKTPKPQPQPQPQPVCSRDFTPPTVKILQSSCDGGGHFPPTIQLLCLVSGYTPGTINITWLEDGQVMDVDLSTASTTQEGELASTQSELTLSQKHWLSDRTYTCQVTYQGHTFEDSTKKCADSNPRGVSAYLSRPSPFDLFIRKSPTITCLVVDLAPSKGTVNLTWSRASGKPVNHSTRKEEKQRNGTLTVTSTLPVGTRDWIEGETYQCRVTHPHLPRALMRSTTKTSGPRAAPEVYAFATPEWPGSRDKRTLACLIQNFMPEDISVQWLHNEVQLPDARHSTTQPRKTKGSGFFVFSRLEVTRAEWEQKDEFICRAVHEAASPSQTVQRAVSVNPGKGSGGGHHHHHHHGGLVPRGSGGGSGRIARLEEKVKTLKAQNSELASTANMLREQVAQLKQKV |
| **RR359(anti-mEGFR) T121G IgE heavy chain ->KiH bsAb** |
| MAVLGLLFCLVTFPSCVLSQVQLQESGGGLVQAGGSLRLSCAASGRTFTSYAMGWFRQVPGKEREFVAALSTRSAGNTYYADSVKGRFTISRDNAKNTVYLQMSSLKAEDTAVYYCAAGYMSSDADPSLAASLHPYDYWGQGTQVTVSSEPKTPKPQPQPQPQPVCSRDFTPPTVKILQSSCDGGGHFPPTIQLLCLVSGYTPGTINITWLEDGQVMDVDLSTASTTQEGELASTQSELTLSQKHWLSDRTYTCQVTYQGHTFEDS**G**KKCADSNPRGVSAYLSRPSPFDLFIRKSPTITCLVVDLAPSKGTVNLTWSRASGKPVNHSTRKEEKQRNGTLTVTSTLPVGTRDWIEGETYQCRVTHPHLPRALMRSTTKTSGPRAAPEVYAFATPEWPGSRDKRTLACLIQNFMPEDISVQWLHNEVQLPDARHSTTQPRKTKGSGFFVFSRLEVTRAEWEQKDEFICRAVHEAASPSQTVQRAVSVNPGKGSGGGHHHHHHH |
| **RR359(anti-mEGFR)IgE heavy chain -> monospecific parental Ab** |
| MAVLGLLFCLVTFPSCVLSQVQLQESGGGLVQAGGSLRLSCAASGRTFTSYAMGWFRQVPGKEREFVAALSTRSAGNTYYADSVKGRFTISRDNAKNTVYLQMSSLKAEDTAVYYCAAGYMSSDADPSLAASLHPYDYWGQGTQVTVSSEPKTPKPQPQPQPQPVCSRDFTPPTVKILQSSCDGGGHFPPTIQLLCLVSGYTPGTINITWLEDGQVMDVDLSTASTTQEGELASTQSELTLSQKHWLSDRTYTCQVTYQGHTFEDSTKKCADSNPRGVSAYLSRPSPFDLFIRKSPTITCLVVDLAPSKGTVNLTWSRASGKPVNHSTRKEEKQRNGTLTVTSTLPVGTRDWIEGETYQCRVTHPHLPRALMRSTTKTSGPRAAPEVYAFATPEWPGSRDKRTLACLIQNFMPEDISVQWLHNEVQLPDARHSTTQPRKTKGSGFFVFSRLEVTRAEWEQKDEFICRAVHEAASPSQTVQRAVSVNPGKGSGGGHHHHHHH |
| **SAM103(anti-mPSMA) T366W KiH IgG1 heavy chain** |
| MAVLGLLFCLVTFPSCVLSEVKLVESEGGLVQPGSSMKLSCTASGFTFSDYYMAWVRQVPEKGLEWVANINYDGTTTYYLDSLKSRFIISRDNSKNILYLQMSSLKSEDTATYYCARVLDGYYGYFDYWGQGTTLSVSSASTKGPSVFPLAPSSKSTSGGTAALGCLVKDYFPEPVTVSWNSGALTSGVHTFPAVLQSSGLYSLSSVVTVPSSSLGTQTYICNVNHKPSNTKVDKKVEPKSCDKTHTCPPCPAPELLGGPSVFLFPPKPKDTLMISRTPEVTCVVVDVSHEDPEVKFNWYVDGVEVHNAKTKPREEQYNSTYRVVSVLTVLHQDWLNGKEYKCKVSNKALPAPIEKTISKAKGQPREPQVYTLPPSRDELTKNQVSL**W**CLVKGFYPSDIAVEWESNGQPENNYKTTPPVLDSDGSFFLYSKLTVDKSRWQQGNVFSCSVMHEALHNHYTQKSLSLSPGKGGSAWSHPQFEK |
| **RR359(anti-mEGFR) T366S, L368A and Y407V KiH IgG1 heavy chain** |
| MAVLGLLFCLVTFPSCVLSQVQLQESGGGLVQAGGSLRLSCAASGRTFTSYAMGWFRQVPGKEREFVAALSTRSAGNTYYADSVKGRFTISRDNAKNTVYLQMSSLKAEDTAVYYCAAGYMSSDADPSLAASLHPYDYWGQGTQVTVSSEPKTPKPQPQPQPQPKTHTCPPCPAPELLGGPSVFLFPPKPKDTLMISRTPEVTCVVVDVSHEDPEVKFNWYVDGVEVHNAKTKPREEQYNSTYRVVSVLTVLHQDWLNGKEYKCKVSNKALPAPIEKTISKAKGQPREPQVYTLPPSRDELTKNQVSL**S**C**A**VKGFYPSDIAVEWESNGQPENNYKTTPPVLDSDGSFFL**V**SKLTVDKSRW  QQGNVFSCSVMHEALHNHYTQKSLSLSPGKGSGGGHHHHHHH |


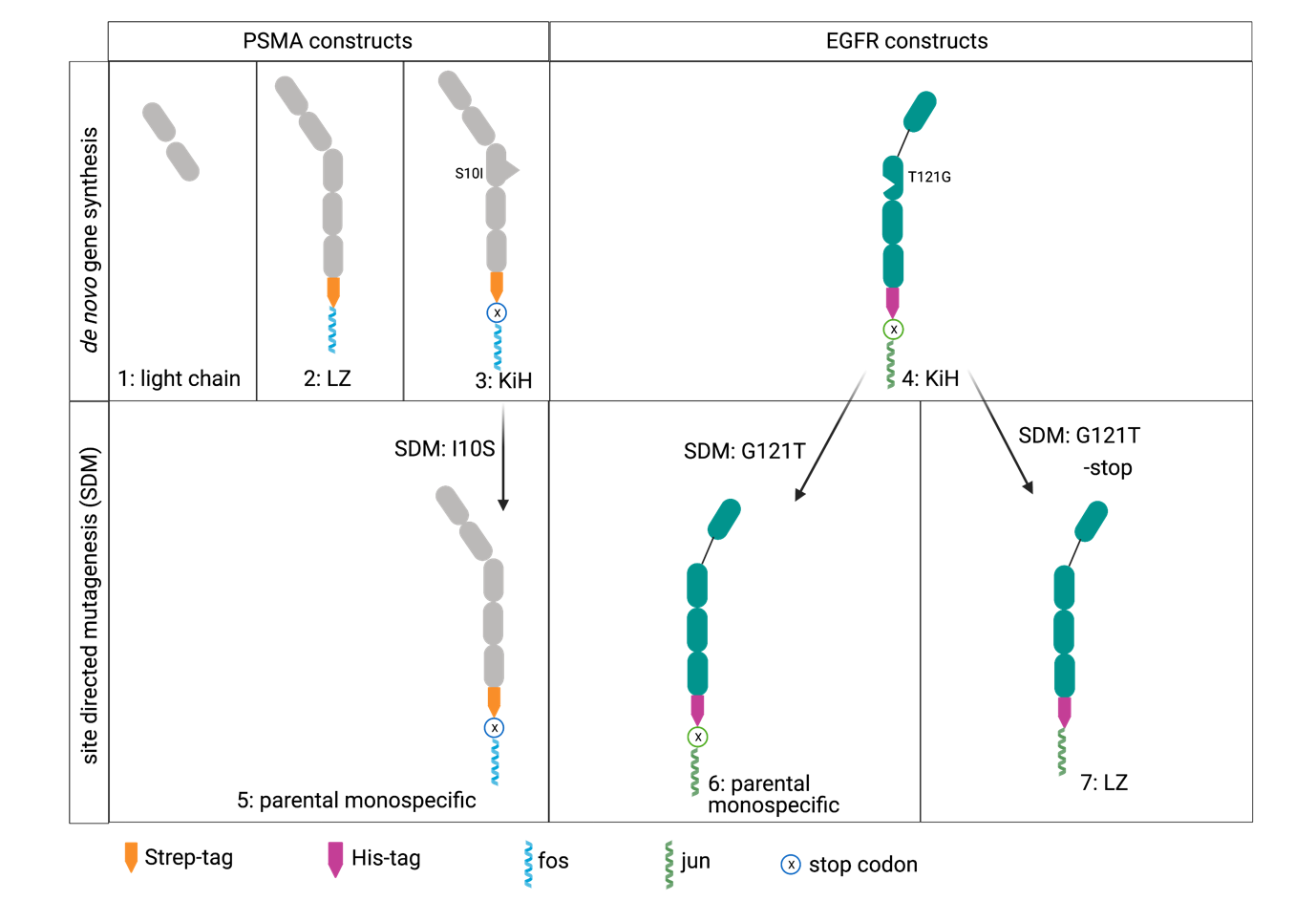


**Supplementary Figure 1.** Antibody DNA constructs were obtained by either *de novo* gene synthesis or site directed mutagenesis.


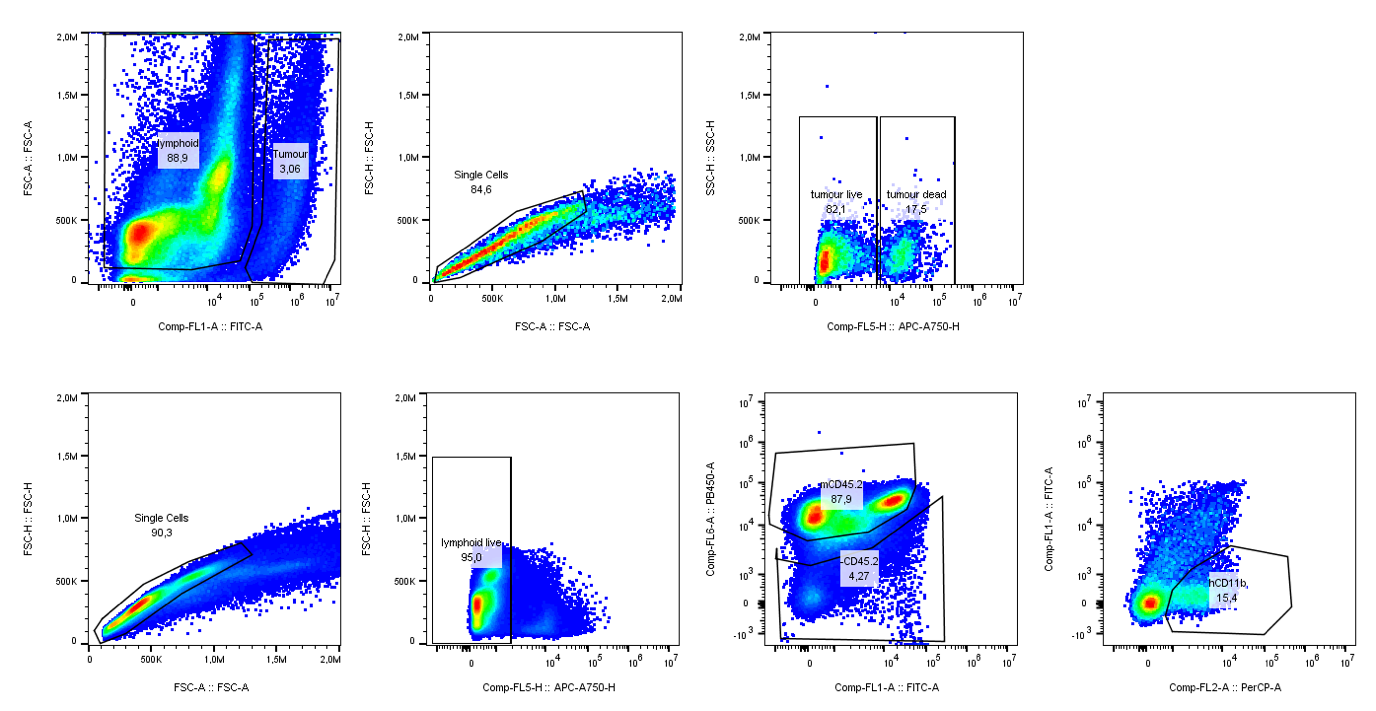


**Supplementary Figure 2. Gating strategy for flow cytometry analysis of peritoneal lavages.**


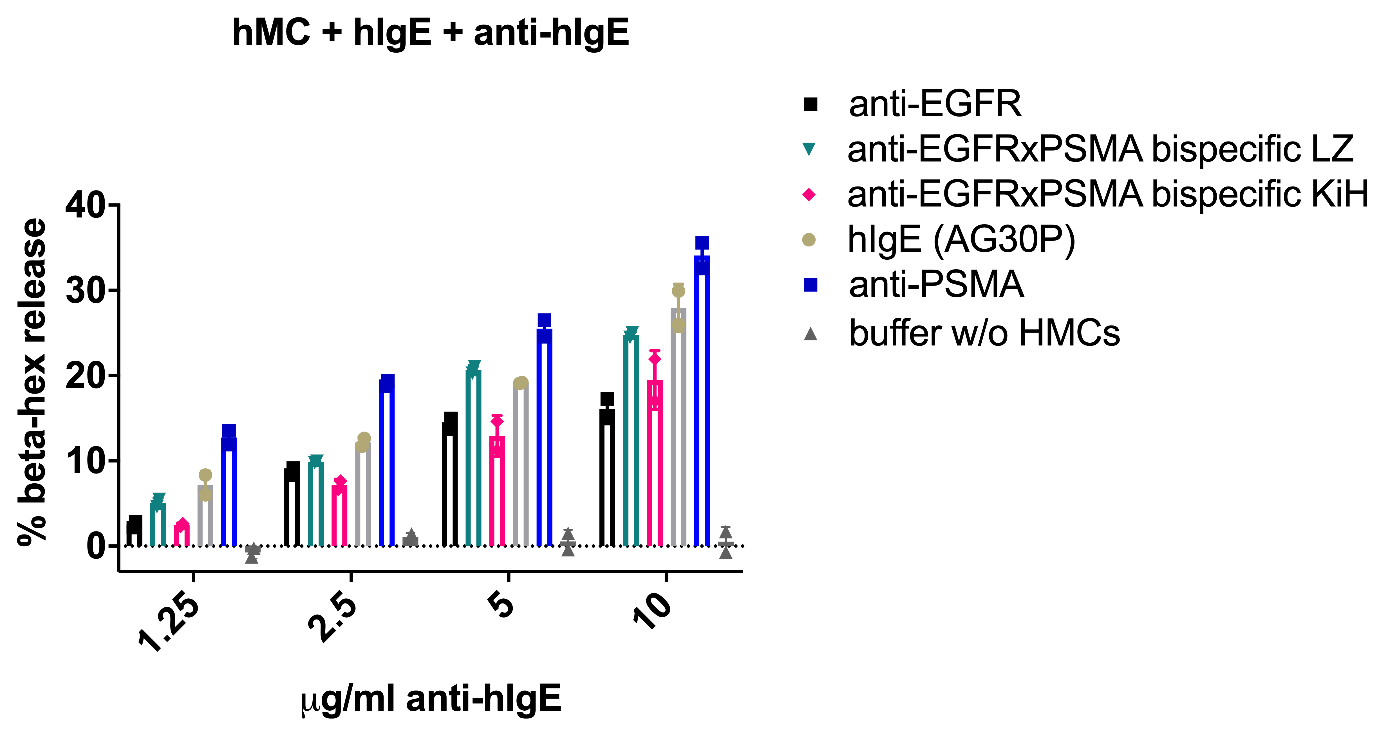


**Supplementary Figure 3. Bispecific IgEs induce mast cell degranulation.** Human mast cells were sensitized with indicated antibodies and the cross-linking was induced with anti-human IgE antibody in a dose-dependent manner.


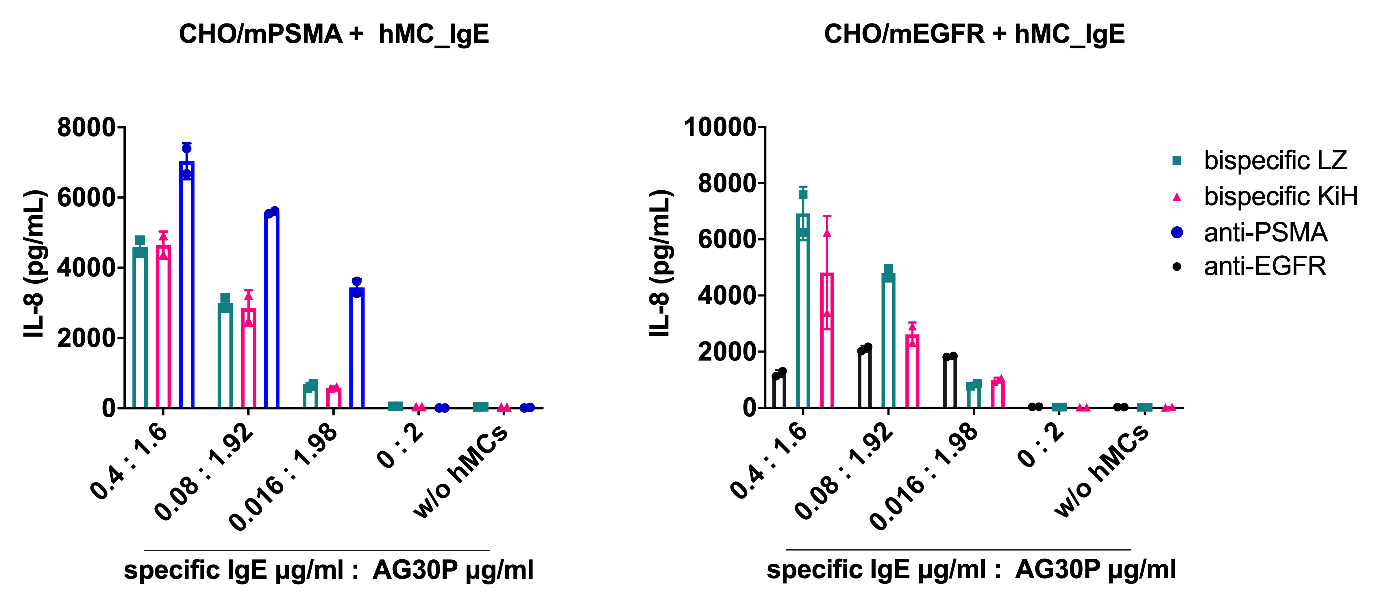


**Supplementary Figure 4. IL-8 levels measured in the supernatant after MC degranulation**


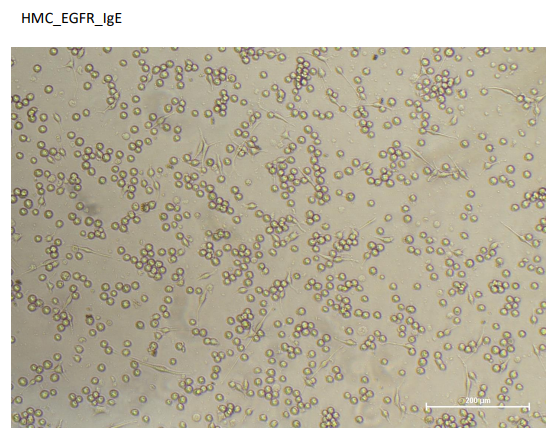


Activated MCs


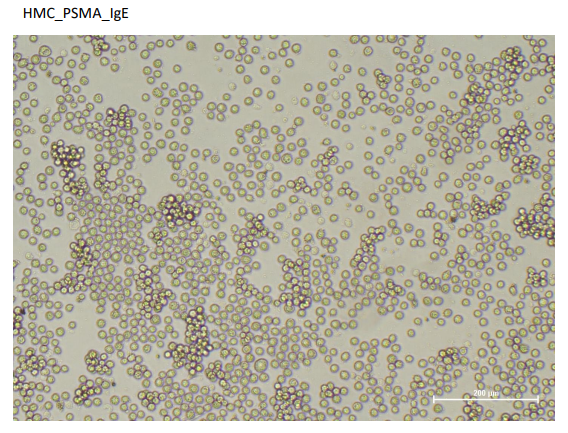


**Supplementary figure 5. MC morphology after sensitization step with indicated IgE antibodies.** Scale bars correspond to 200μm.
